# Is There an Interplay Between the Functional Domains of IRAP?

**DOI:** 10.3389/fcell.2020.585237

**Published:** 2020-09-29

**Authors:** Anika Vear, Tracey Gaspari, Philip Thompson, Siew Yeen Chai

**Affiliations:** ^1^Department of Physiology, Monash Biomedicine Discovery Institute, Monash University, Clayton, VIC, Australia; ^2^Department of Pharmacology, Monash Biomedicine Discovery Institute, Monash University, Clayton, VIC, Australia; ^3^Department of Medicinal Chemistry, Monash Institute of Pharmaceutical Sciences, Monash University, Parkville, VIC, Australia

**Keywords:** IRAP, Angiotensin IV, M1 aminopeptidase, pathophysiology, trafficking, IRAP inhibitors

## Abstract

As a member of the M1 family of aminopeptidases, insulin regulated aminopeptidase (IRAP) is characterized by distinct binding motifs at the active site in the C-terminal domain that mediate the catalysis of peptide substrates. However, what makes IRAP unique in this family of enzymes is that it also possesses trafficking motifs at the N-terminal domain which regulate the movement of IRAP within different intracellular compartments. Research on the role of IRAP has focused predominantly on the C-terminus catalytic domain in different physiological and pathophysiological states ranging from pregnancy to memory loss. Many of these studies have utilized IRAP inhibitors, that bind competitively to the active site of IRAP, to explore the functional significance of its catalytic activity. However, it is unknown whether these inhibitors are able to access intracellular sites where IRAP is predominantly located in a basal state as the enzyme may need to be at the cell surface for the inhibitors to mediate their effects. This property of IRAP has often been overlooked. Interestingly, in some pathophysiological states, the distribution of IRAP is altered. This, together with the fact that IRAP possesses trafficking motifs, suggest the localization of IRAP may play an important role in defining its physiological or pathological functions and provide insights into the interplay between the two functional domains of the protein.

## Introduction

**FIGURE 1:**
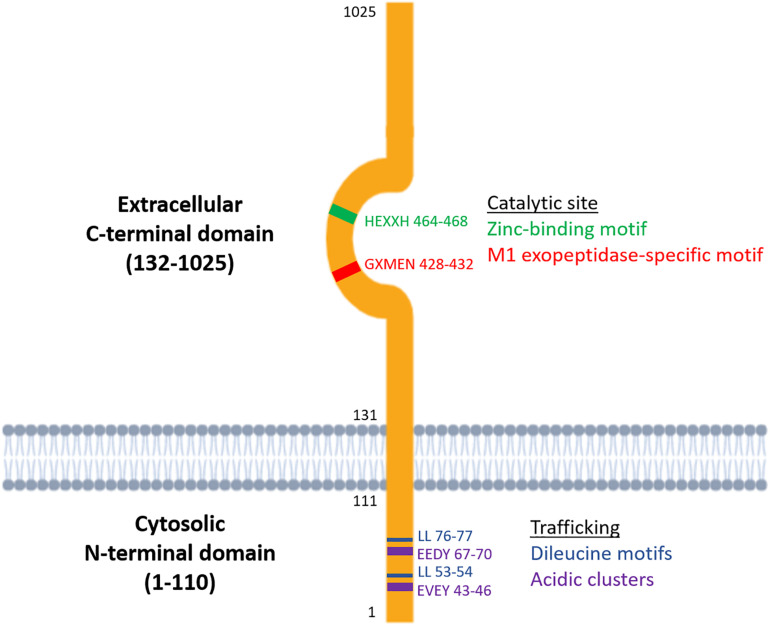
Structure of the two functional domains of IRAP: the cytosolic N-terminal domain which contains trafficking motifs and the extracellular C-terminal domain where the catalytic site is found.

Metallopeptidases are a diverse family of proteolytic enzymes which are involved in regulating the activity of peptide hormones that play crucial roles in maintaining homeostatic balance in physiology (Cerda-Costa and Gomis-Ruth, 2014). Failure of these regulatory mechanisms has been shown to result in pathologies such as inflammation, tissue/organ dysfunction, neurological diseases and cardiovascular disorders (Lorenzl et al., 2003; Libby, 2006). A sub-family of metallopeptidases, the metallo-type 1 (M1) aminopeptidases, have important functions in cell maintenance, defense, and growth and development (Drinkwater et al., 2017). Structurally, the M1-aminopeptidases are characterized by two distinct binding motifs, the HEXXH zinc-binding motif which is involved in catalysis and the GXMEN exopeptidase motif which is an N-terminal recognition site that confers selectivity for peptide substrates (Figure 1; Cerda-Costa and Gomis-Ruth, 2014). These highly conserved motifs are found at the active site in the C-terminal domain and mediate the catalysis of a range of peptide substrates. This family consists of nine enzymes in humans, six of which are integral membrane proteins and three of which are found in the cytoplasm (Table 1). Interestingly, only one of these M1 aminopeptidases has a large cytosolic N-terminus which possesses motifs that regulate trafficking events (Figure 1; Keller et al., 1995). This enzyme, known as insulin regulated aminopeptidase (IRAP), is the focus of the current review.

**TABLE 1:** Enzymes in the M1 aminopeptidase family.

**M1 aminopeptidases**	**Size of protein in humans (number of amino acids)**	**Size of cytosolic N-terminal domain in humans (number of amino acids)**	**UniProt ID**
**Membrane-bound enzymes**			
Aminopeptidase A	957	18	Q07075
Thyrotropin-releasing hormone-degrading ectoenzyme	1,024	40	Q9UKU6
Aminopeptidase N	967	7	P15144
Endoplasmic-reticulum aminopeptidase 1 (ERAP1)	941	1	Q9NZ08
Endoplasmic-reticulum aminopeptidase 2 (ERAP2)	960	20	Q6P179
Insulin regulated aminopeptidase (IRAP)	1,025	110	Q9UIQ6
**Cytoplasmic enzymes**			
Aminopeptidase B	650	–	Q9H4A4
Puromycin-sensitive aminopeptidase	919	–	P55786
Leukotriene A4 hydrolase	611	–	P09960

Much of the earlier work on elucidating the enzymatic role of IRAP have involved the use of angiotensin IV (Ang IV), a peptide inhibitor. Treatment with Ang IV or other IRAP inhibitors elicited effects on facilitation of memory (Braszko et al., 1988; Wright et al., 1993; Albiston et al., 2008; De Bundel et al., 2009; Royea et al., 2019) and protection against ischemic damage (Coleman et al., 1998; Faure et al., 2006a; Park et al., 2016). It can be reasonably asserted that these peptide inhibitors bind to IRAP at the cell surface, as like many M1 aminopeptidases, IRAP is an integral membrane protein with its catalytic domain on the cell exterior. This enables the regulation of the levels of circulating or extracellular peptide substrates that are secreted to exert autocrine, paracrine and endocrine activities. Given the unique role of the N-terminus of IRAP in the regulation of vesicular trafficking and tethering, the subcellular location of the protein is also an important consideration when investigating its function. Additionally, the mobilization of IRAP to the plasma membrane is observed in various disease settings suggesting there is an increased demand for its catalytic activity at the cell surface in these states. These properties of IRAP suggest that there is an interplay between the two functional domains of the enzyme and that its spatial location in the cell has important implications in the temporal determination of its aminopeptidase activity. This review will examine our current understanding of the physiological roles of the C- and N-terminus of IRAP and discuss potential evidence of an interplay between these domains.

## Targeting the C-Terminal Domain of IRAP

### Peptide Substrates of IRAP Inform on Its Function

The most extensively studied function of IRAP is the ability of its catalytic C-terminus to cleave a range of substrates albeit *in vitro*. These substrates include oxytocin, vasopressin, lys-bradykinin, angiotensin III, met-enkephalin, dynorphin A 1–8, neurokinin A, neuromedin B, somatostatin, and cholecystokinin 8 (Matsumoto et al., 2001a). There is great diversity in the structure of these substrates, from large cyclic peptides such as oxytocin and vasopressin to small linear peptides like met-enkephalin. This diversity can be attributed to the plasticity of the active site of IRAP. For example, remodeling of the GXMEN exopeptidase loop and reorientation of the key aromatic residue, Tyr549, results in an increased active site volume of ∼5,300 Å compared with ERAP1’s closed state volume of 2,920 Å and provides a potential explanation for IRAP’s ability to bind large cyclic substrates such as oxytocin and vasopressin (Hermans et al., 2015). This broad substrate specificity suggests IRAP is a relatively promiscuous enzyme and that potentially its conformation and subcellular localization may inform on substrate selectivity.

The diverse physiological roles of the substrates of IRAP range from blood pressure control to the pain response to digestion, reflecting the importance of the enzyme in regulating peptide hormone levels across multiple endocrine and paracrine systems. Notably, only vasopressin has been identified as a physiological substrate of IRAP *in vivo*. This was done by comparing the clearance of radiolabeled vasopressin from the circulation of wildtype and IRAP knockout mice. At 5 min after injection of [^125^I]-vasopressin, there was a ∼4-fold decrease in the intact substrate in the plasma of wildtype mice compared with IRAP knockout mice, with no intact substrate detected in wildtype mice after 20 min (Wallis et al., 2007). These findings were validated with the N-terminal cleavage product, 3-iodo-[^125^I]tyrosine, detectable only in plasma from wildtype and not knockout mice. In conjunction with the observations that IRAP knockout mice compensate for increased circulating vasopressin levels by decreasing vasopressin synthesis, it was concluded that IRAP plays a role in the cleavage and clearance of vasopressin from the circulation (Wallis et al., 2007).

It has also been proposed that one of the physiological roles of IRAP is regulating levels of circulating oxytocin, a peptide hormone important for initiating contractions during labor (Uvnas-Moberg et al., 2019). Interestingly, oxytocin was suggested to be degraded by secreted soluble IRAP rather than its membrane bound form (Rogi et al., 1996). Northern blot analysis revealed increasing levels of IRAP mRNA in human placental tissue with gestation, peaking at 38 weeks, which correlates with the increases in circulating oxytocin in the latter stages of pregnancy (Yamahara et al., 2000). Thus, it was proposed that oxytocin degradation by IRAP regulates the onset of labor in humans. However, there is conflicting evidence when we look at the reproductive profiles of IRAP KO mice. In support of the role of IRAP in regulating pregnancy, a significant shortening of gestational term was observed in IRAP KO mice compared with wildtype controls which was further shortened following administration of oxytocin (Ishii et al., 2009). In contrast, Pham et al. (2009) found IRAP deficient mice had no apparent differences in gestational length compared with wildtype controls. It was acknowledged by these authors that the Phe154-Ala155 cleavage site is absent in mice and many other species. Therefore, the role of circulating IRAP during pregnancy is likely restricted to humans. It is also possible that other serum enzymes inactivate oxytocin, with oxytocin cleared from circulation *in vivo* in the absence of IRAP (Wallis et al., 2007). Given there is ample evidence on this area in humans and that soluble IRAP has not been extensively characterized, it is too early to conclude that one of the physiological roles of IRAP is in oxytocin regulation during pregnancy.

Another physiological role of IRAP focuses on its aminopeptidase activity in dendritic cells where IRAP and its close family members, ERAP1 and 2, participate in antigen trimming, a process which is crucial in regulating the presentation of antigen epitopes onto MHC class I molecules in the adaptive immune response. This process was clearly demonstrated in a study by Saveanu et al., 2009, where IRAP-deficiency in bone-marrow derived dendritic cells resulted in a reduction of antigen presentation by 50–70% compared with wild type controls. Unlike ERAP1/2, IRAP is involved in the alternative endosomal pathway, in which exogenous antigens are directly loaded onto MHC class I molecules in endosomes as opposed to the endoplasmic reticulum (Joffre et al., 2012). Thus, IRAP as the principal trimming aminopeptidase in endosomes, is postulated to play a crucial role in cross presentation. Notably, this is the only known example in which IRAP is catalytically active intracellularly and suggests the spatial demand for its aminopeptidase activity may be cell- and tissue-dependent.

Various studies have also probed for IRAP’s substrate preference in terms of amino acid length and sequence, by measuring its ability to trim antigenic peptide precursors. Similar to ERAP1, IRAP was able to efficiently trim a variety of long peptide sequences and accumulate mature antigenic epitopes of 8 or 9 amino acids (Georgiadou et al., 2010). However, in contrast to ERAP1, IRAP was more efficient in accumulating smaller products by further trimming of the mature antigenic epitopes (Georgiadou et al., 2010). These findings suggest IRAP has a broad substrate selectivity compared with ERAP1 and is able to process peptide substrates of varying lengths and amino acid side chains.

### Angiotensin IV, a Competitive Inhibitor of IRAP

IRAP has been identified as the angiotensin type 4 receptor (AT_4_R), the specific and high affinity binding site for Ang IV, a hexapeptide corresponding to residues 3–8 of angiotensin II (Ang II) (Albiston et al., 2001). This conclusion was supported by evidence that membranes from cells transfected with the full-length cDNA for human IRAP crosslinked with a photoactivable analog of Ang IV, [^125^I]Nle1-BzPhe6-Gly7-Ang IV. SDS-PAGE revealed the crosslinked IRAP was resolved as a major band of 165 kDa which was displaceable by 10 μM Ang IV, observations consistent with the previously characterized AT4R (Albiston et al., 2001). Additionally, *in vitro* receptor autoradiography confirmed the localization of both IRAP mRNA and protein in mouse brain sections correlated with the distribution of the AT_4_R (Albiston et al., 2001). These findings, along with a loss of Ang IV binding to the AT_4_R in the brain of the IRAP knockout mouse, provided compelling evidence that IRAP is the AT_4_R.

Ang IV was first shown to bind to IRAP in bovine adrenal cortex membranes (Swanson et al., 1992). Additionally, IRAP also binds LVV-hemorphin-7 (LVV-H7) (Moeller et al., 1997), which is structurally distinct and shares little sequence homology with Ang IV (Moeller et al., 1999a). Unlike substrates of the enzyme, both Ang IV and LVV-H7 are resistant to proteolytic degradation by IRAP and instead were found to be competitive inhibitors of IRAP. This was shown by Lew, Mustafa (28) where the inhibitory effects of the IRAP ligands Ang IV, Nle^1^-Ang IV, divalinal-Ang IV, and LVV-H7 was assessed following cleavage of leu-β-naphthylamide (Leu-β-NA), a synthetic substrate of the enzyme. All four of the tested IRAP ligands were seen to inhibit IRAP activity, with Ang IV displaying the highest potency (*K*_*i*_ = 113 nM) (Lew et al., 2003). Lineweaver Burk analysis also revealed a 14-fold increase in the K_*m*_ for Leu-β-NA with increasing inhibitor concentrations and only minor changes in V_*max*_. Thus, it was concluded that IRAP inhibitors display competitive kinetics, indicating they bind directly to the catalytic site of the enzyme (Lew et al., 2003).

Despite Ang IV and LVV-H7 binding with high affinity to IRAP to inhibit its catalytic activity, a number of factors have hindered their potential for therapeutic use and as research tools to examine IRAP function. These include their limited stability in the circulation and lack of specificity for IRAP over other aminopeptidases and receptors such as the AT1R for Ang IV and μ-opioid receptor for LVV-H7 (Lew et al., 2003). Thus, a range of novel peptidomimetic (Axen et al., 2007; Lukaszuk et al., 2008; Andersson et al., 2010; Kokkala et al., 2016) and small molecule inhibitors (Albiston et al., 2008; Borhade et al., 2014) have been developed to address these limitations with some of these compounds showing promise as potential therapeutic agents.

### IRAP Inhibition Facilitates Learning and Memory

Whilst Ang IV was initially thought to just be an inactive metabolic fragment of Ang II, it was demonstrated to have memory-enhancing properties in rats and mice in a range of memory and cognitive tasks. This facilitation of learning and memory occurs following acute central administration of Ang IV (Braszko et al., 1988; Wright et al., 1993), LVV-H7 (De Bundel et al., 2009), as well as with the small molecular weight IRAP inhibitor, HFI-419 (Albiston et al., 2008), further strengthening the proposal that inhibition of IRAP leads to enhancement of memory. IRAP therefore presents as a promising target for neurodegenerative diseases such as Alzheimer’s Disease (AD), in which there is progressive cognitive decline and memory loss. This was validated in a recent study by Royea et al. (2019) in which 4 weeks of centrally administered Ang IV (1.3 nmol/day) restored short-term memory, spatial learning and spatial memory in a transgenic mouse model of AD with alternatively spliced amyloid precursor protein.

Evidence of changes in the brain with IRAP inhibitor treatment include normalization of hippocampal subgranular zone cellular proliferation, enhanced dendritic branching, and reduced oxidative stress (Royea et al., 2019). Furthermore, treatment of primary rat hippocampal cultures with a number of different classes of IRAP inhibitors has been shown to significantly alter dendritic spine morphology and increase spine density (Diwakarla et al., 2016a,b; Seyer et al., 2019). Similarly, enhancement of long-term potentiation was observed in rats in the dentate gyrus *in vivo* following Ang IV treatment (Wayner et al., 2001) and within the hippocampus *in vitro* following treatment with Ang IV analogs (Kramar et al., 2001). The changes in spine morphology are thought to correlate with the strength and activity of the synapse, which coupled with enhanced long-term potentiation, provides strong supporting evidence of the memory enhancing role of IRAP inhibitors.

Despite these effects of IRAP inhibitors on memory enhancement, Albiston et al. (2010) found that global deletion of the IRAP gene resulted in mice with an accelerated, age-related decline in spatial memory as seen in the Y maze task. Similarly, conditional forebrain neuron specific-IRAP knockout led to deficits in spatial reference and novel object recognition memory (Yeatman et al., 2016). These unexpected observations were thought to potentially be due to altered brain development of the knockout mice, given there is high IRAP expression in the highly neurogenic ventricular and subventricular zone of the embryonic mouse brain, possibly implicating IRAP as having a role in neuronal development. Regardless of this finding, there is comprehensive evidence supporting the cognitive enhancing effect of IRAP inhibition (Albiston et al., 2004; Lee et al., 2004; De Bundel et al., 2009; Fidalgo et al., 2019; Royea et al., 2019).

Ang IV has also been shown to have positive effects on other central nervous system functions. For example, centrally administered Ang IV (0.1–1 μg/mouse) provides protection against pentylenetetrazol (PTZ)-induced seizures in mice, by dose-dependently increasing PTZ-seizure threshold and decreasing PTZ-seizure intensity (Tchekalarova et al., 2001). Additionally, inhibition of IRAP by Ang IV has an anxiolytic (Beyer et al., 2010) and anti-hyperalgesia (Chow et al., 2013) effect in rats and an anti-allodynia effect in male mice (Chow et al., 2018), all proposed to be due to elevated levels of oxytocin in the brain or spinal cord.

### IRAP Inhibitor Treatment Protects Against Ischemic Damage

IRAP also presents as a promising target for the treatment of ischemic damage in the brain and heart. For example, IRAP inhibition with Ang IV (1 nmol) in a rat model of embolic stroke has been shown to significantly reduce cerebral infarct volume compared with untreated controls (Faure et al., 2006a). Importantly, this reduction corresponded with improvements in neurological performance and decreased mortality at 24 h post-stroke and is likely attributed to redistribution of blood flow to the ischemic areas (Faure et al., 2006a). However, these initial findings on the vasodilatory effect of Ang IV was contradicted by a more recent study by Faure et al. (2006b) who suggested low concentrations of Ang IV (0.001–3 μM) had a vasoconstrictive effect on isolated rat basilar arteries that is mediated by binding to IRAP. Given that increases in blood flow to damaged brain areas with subsequent neurological improvement have been consistently reported in a number of studies, it is likely that Ang IV acts as a vasodilator in the brain.

There are conflicting reports on the effects of Ang IV on the renal vasculature as well. Interestingly, this effect appears to be dependent on the concentration and route of administration of the inhibitor, with infusion of low doses of Ang IV (0.1–100 pmol) directly into renal arteries increasing renal blood flow (Coleman et al., 1998), compared with systemic administration of high concentrations of Ang IV (100 nmol/kg) which results in renal vasoconstriction (Fitzgerald et al., 1999; Yang et al., 2008). At higher concentrations, Ang IV also binds to the AT_1_R which may mediate this vasoconstrictory effect. Therefore, lower concentrations of Ang IV (<1 μM) should be used to investigate the effect of IRAP inhibition to avoid off-target binding. Similarly in the heart, 3 days of pre-treatment with Ang IV (1 mg/kg/day) in rats was found to blunt the ischemia/reperfusion (I/R)-induced increases in pro-fibrotic factors such as TNF-α, MMP-9, and VCAM-1 (Park et al., 2016). This led to an overall improvement in I/R-induced myocardial dysfunction in these rats. In combination, these studies suggest that loss of function of the catalytic activity of IRAP provides protection against ischemic events in multiple organs, which may potentially also prevent downstream consequences of this ischemic damage such as tissue fibrosis.

### IRAP Inhibition Is Cardio- and Vaso-Protective

Ang IV treatment has also been observed to be cardio-protective, displaying opposing effects to Ang II. Yang et al. (2011) demonstrated that Ang II-induced cardiac dysfunction and injury were improved following Ang IV administration (100 nM) in isolated rat heart. Ang IV was also seen to inhibit Ang-II induced cell apoptosis, cardiomyocyte hypertrophy, and collagen synthesis of cardiac fibroblasts (Yang et al., 2011). Following IRAP siRNA knockdown, it was concluded that these effects were mediated by IRAP (Yang et al., 2011). The vaso-protective actions of Ang IV have also been observed, with increased aortic nitric oxide bioavailability following chronic Ang IV treatment (1.44 mg/kg/day) of high-fat diet fed apolipoprotein E-deficient mice (Vinh et al., 2008b). This improvement was associated with decreased superoxide levels and increased endothelial nitric oxide synthase-3 expression. Similarly, treatment with the small molecule IRAP inhibitor, HFI-419, prevents against acetylcholine-mediated vasoconstriction *in vitro*, in abdominal aorta from a rabbit model of coronary artery vasospasm (El-Hawli et al., 2017). These studies, in conjunction with those mentioned earlier on IRAP in I/R injury, suggest that IRAP inhibition is cardio- and vaso-protective.

### Knowledge Gaps

In summary, the catalytic activity of the C-terminal domain of IRAP has been shown to have important functions in many body systems, ranging from reproduction to the cardiovascular system. Interestingly, whilst it is required for maintaining normal physiological processes in some of these systems, its upregulation is seen in many pathophysiological states such as atherosclerosis (Vinh et al., 2008a), following balloon injury of the carotid arteries (Moeller et al., 1999b) and in response to hypoxia in the carotid body (Fung et al., 2007), suggesting the consequences of its functions are cell/tissue- and state-dependent. Generally, inhibition of IRAP appears to provide protection against many of these diseases, encouraging researchers to further validate IRAP as a promising therapeutic target. However, many questions remain regarding the specific mechanisms underlying IRAP’s role in disease, and whether it is a cause or consequence of disease pathogenesis. Better understanding of these mechanisms would also provide important insights to guide the design of novel IRAP inhibitors as therapeutic agents. Notably, the subcellular localization of IRAP, regulated by its other functional domain at the N-terminus, has not been investigated in many of these contexts, nor has the trafficking of IRAP following inhibitor binding. It is also not known if the current classes of IRAP inhibitors have access to the catalytic site of IRAP in its location in intracellular vesicles. Interestingly, it has been demonstrated that the distribution of IRAP is altered in various disease states. Collectively, these findings suggest the efficacy of IRAP inhibitors may be influenced by the location of the enzyme. The trafficking of IRAP may reflect or regulate its function at a given point in time and location. Therefore, future research recognizing the role of the N-terminal domain and its potential influence on IRAP’s catalytic activity may help us to better understand IRAP function in disease.

## Recognising the N-Terminal Domain of IRAP

### Trafficking Motifs at the N-Terminus of IRAP Regulate Its Movement in the Cell

The cytosolic N-terminus is unique to IRAP compared with other M1 aminopeptidases and contains two dileucine motifs, each with a preceding acidic cluster (Keller et al., 1995). This domain is postulated to have two key functions both of which influence the subcellular distribution of IRAP. One of these involves regulating the trafficking of IRAP in vesicles in turn, modulating the subcellular distribution of associated vesicular proteins. The key studies exploring the trafficking of IRAP have been conducted in 3T3-L1 adipocytes, following observations that IRAP colocalized with the insulin responsive glucose transporter, glucose transporter isoform 4 (GLUT4), in these cells (Keller et al., 1995). This was clearly demonstrated by Waters et al. (1997), where microinjection of a fusion protein containing the cytosolic domain of IRAP into 3T3-L1 adipocytes resulted in translocation of GLUT4-containing vesicles to the plasma membrane. Interestingly, this increase in cell surface GLUT4 was equivalent to that seen following stimulation with insulin and was not blocked by wortmannin, an inhibitor of the PI3K signaling pathway downstream of insulin receptor activation (Waters et al., 1997). This suggests the translocation of GLUT4 was regulated by the N-terminus of IRAP. The fact that IRAP, unlike its close family members, has these trafficking motifs suggests that tight regulation of its localization must be important to, or even regulate, its function.

**FIGURE 2:**
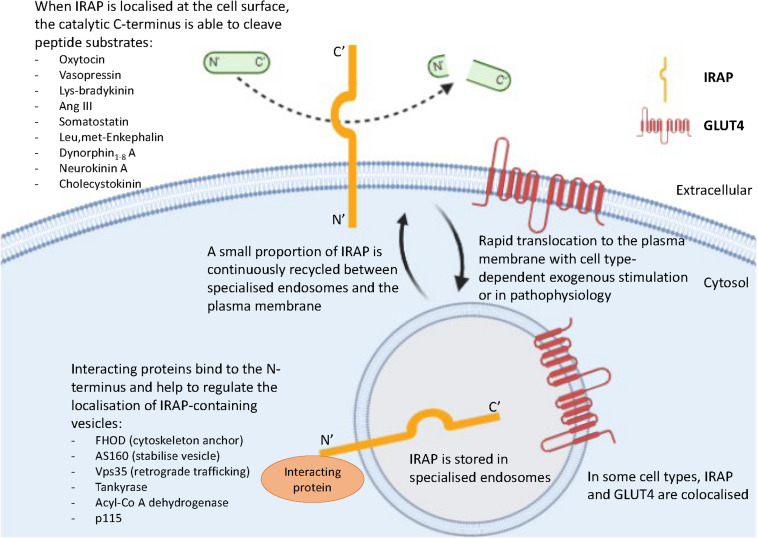
Key roles of the two functional domains of IRAP. IRAP is predominantly localized in specialized endosomes with a small proportion of IRAP-containing vesicles slowly recycling to the plasma membrane. This movement is regulated by the binding of interacting proteins to the N-terminal domain of IRAP. At the plasma membrane, the catalytic C-terminus of IRAP is exteriorized and therefore has access to cleave a range of peptide substrates.

In the basal state, newly synthesized IRAP is targeted to the trans-Golgi network (TGN), evidenced by colocalization with a TGN-specific marker (TGN38) (De Bundel et al., 2015). It is subsequently transported for storage to specialized endosomal vesicles in the perinuclear region of the cell, with slow recycling of these endosomes to the plasma membrane (Figure 2; Shi et al., 2008; Jordens et al., 2010). The dileucine motif at position 76 and 77 of IRAP’s cytoplasmic N-terminal domain was found to be required for this sorting to specialized endosomes in adipocytes, with alanine substitution of these residues resulting in a dramatic increase in the cell surface localization of IRAP (Hou et al., 2006). Notably, an electron microscopy study in hippocampal neurons found IRAP specific immunoreactivity predominantly associated with 100–200 nm vesicles (Fernando et al., 2007). This finding, along with those from sub-cellular fractionation and dual-label immunofluorescence experiments, led the authors to propose the subcellular distribution of IRAP in these neurons is very similar to that seen in insulin-responsive cells (Fernando et al., 2007). Thus, the trafficking of IRAP observed in adipocytes can likely be generalized to other cell types such as neurons and cardiac fibroblasts, in which there is a distinct perinuclear IRAP distribution in a basal state.

### IRAP Has a Role in Tethering Vesicles

The N-terminus of IRAP is also believed to be important for tethering specialized endosomes to the cytoskeleton via binding of interacting proteins including tankyrase (Chi and Lodish, 2000), acyl-coenzyme A dehydrogenase (Katagiri et al., 2002), formin homology domain (FHOD) (Babdor et al., 2017), p115 (Hosaka et al., 2005), Vps35 (Pan et al., 2019), and Akt substrate of 160 kDa (AS160) (Figure 2; Peck et al., 2006). This has been clearly demonstrated in dendritic cells, in which the role of the N-terminal domain of IRAP has only recently been implicated in immune function. A comprehensive study by Babdor et al. (2017) revealed that anchorage of endosomes containing toll-like receptor 9 (TLR9) to the actin cytoskeleton by the N-terminus of IRAP via the interacting protein, FHOD4, prevents TLR9 activation. Thus, in the absence of IRAP, the trafficking of TLR9 to lysosomes and subsequent activation was enhanced in cultured dendritic cells and in mice following bacterial infection, leading to an increased secretion of pro-inflammatory cytokines (Babdor et al., 2017). This study is crucial as it not only suggests another intracellular trafficking mechanism regulated by IRAP, but also has potential implications for understanding the mechanisms underlying certain autoimmune disorders such as arthritis, where there is inappropriate activation of endosomal TLRs (Rifkin et al., 2005). Furthermore, the importance of IRAP in immune functioning is supported by the identification of a deleterious missense mutation in IRAP’s gene associated with psoriasis, an autoimmune disorder linked to activation of TLR9 (Cheng et al., 2014).

The role of IRAP in tethering vesicles is also seen in adipocytes in which it is thought to regulate GLUT4 translocation. For example, Peck et al. (2006) proposes that a GTP-ase activating protein (GAP), AS160, is tethered with the cytosolic tail of IRAP in the basal state thus preventing the translocation of GLUT4 storage vesicles (GSVs). Subsequent stimulation by insulin is suggested to inactivate AS160, resulting in translocation of the GSV to the plasma membrane, thus facilitating glucose uptake by the cell. Notably, immunostaining revealed IRAP, GLUT4 and AS160 all have a punctate-like expression in the cytoplasm in a basal state, with areas of concentration adjacent to the nucleus, whilst insulin stimulation caused a marked redistribution of IRAP and GLUT4 to the plasma membrane (Peck et al., 2006). A recent study also found retrograde trafficking of IRAP from endosomes to the TGN was dependent on binding of the retromers Vps35 and Vps26 to the cytoplasmic tail of IRAP (Pan et al., 2019). Not only did these retromers strongly colocalize with IRAP in unstimulated 3T3-L1 adipocytes, but knockout of Vps35 in particular, increased the targeting of IRAP to lysosomes due to the lack of retromer-dependent endosomal retrieval to the TGN (Pan et al., 2019).

Studies exploring the functions of the N-terminus of IRAP in trafficking and tethering specialized vesicles have shed new light on its potential roles in these cells. However, the physiological relevance of these functions is not well-understood. Despite this, the fact that IRAP possesses a cytosolic N-terminus which has the ability to not only regulate its trafficking but that of other vesicular proteins, suggests the subcellular localization of IRAP must be significant in regulating its function. This logic provides the first hint of an interplay between IRAP’s functional domains.

## An Interplay Between the Functional Domains of IRAP?

### Subcellular Localization of IRAP Is Altered in Pathophysiology

In a basal state IRAP is predominantly located intracellularly in specialized endosomes, with slow recycling of these vesicles to the plasma membrane (Shi et al., 2008; Jordens et al., 2010). However, this recycling appears to be altered in pathophysiology. For example, following exogenous stimulation, IRAP is seen to rapidly translocate to the cell surface. Measurement of cell surface IRAP using a cell surface biotinylation method revealed that following treatment of 3T3-L1 adipocytes with insulin, there was an 8-fold increase in the proportion of IRAP at the plasma membrane (Ross et al., 1998). Similar results were seen in the same cell line using an alternative technique; immunofluorescence with an IRAP specific antibody (Liu et al., 2005). Redistribution of IRAP to the cell surface was also observed following stimulation with endothelin-1 in 3T3-L1 adipocytes (Wu-Wong et al., 1999), oxytocin in human vascular endothelial cells (Nakamura et al., 2000), immunoglobulin E in mast cells (Liao et al., 2006) and forskolin in PC12 cells (Matsumoto et al., 2001b). This translocation of IRAP is potentially indicative of an increased cellular demand for the C-terminus of IRAP to be expressed at the cell surface in these stimulated states to subserve a particular protective or pathophysiological role. Importantly, some of these stimulants mimic what is seen in various pathophysiological settings in which IRAP has a predominantly cell surface localization. For example, an increase in cell surface IRAP, measured by binding of the radiolabeled IRAP inhibitor, [H^3^]IVDE77, was observed in mouse pro-inflammatory M1-activated macrophages following stimulation with interferon-γ or lipopolysaccharide (Nikolaou et al., 2014).

Furthermore, an alteration to the characteristic recycling of IRAP is seen in type 2 diabetes. Maianu et al. (2001) examined the subcellular localization of both GLUT4 and IRAP in adipocytes isolated from healthy controls and type 2 diabetics and found that despite no detectable difference in IRAP protein expression between groups, IRAP and GLUT4 trafficking in diabetic patients appeared to be altered with redistribution to high-density microsomes and plasma membrane fractions in basal cells and impaired translocation following insulin stimulation. This results in impaired insulin action and a decrease in insulin-responsive vesicular trafficking (Maianu et al., 2001). Importantly, the accumulation of IRAP at the plasma membrane in type 2 diabetics correlates with the redistribution of IRAP to the cell surface in other disease states.

Overall, these studies suggest that the slow recycling of IRAP-containing vesicles to the plasma membrane seen in a basal state, is altered in pathophysiology, and mimics what is reported following exogenous stimulation. This potentially provides a mechanism by which the cell is able to regulate IRAP function by altering its localization. It is also worth considering whether this change in IRAP distribution contributes to the disease phenotype, with both a loss of IRAP in one location and a gain of IRAP in another having positive or negative consequences on the cell.

### Physiological Example of IRAP Trafficking Regulating Its Catalytic Activity

Most studies to date have explored the functions of either the C- or N-terminus of IRAP independently. However, there is one reported physiological example in which a clear relationship between these domains was demonstrated. In the study by Wallis, Lankford (16), fasted wildtype or IRAP knockout mice were injected with saline or insulin, and then 5 min later, with radiolabeled vasopressin. Blood samples taken at 1, 2, and 3 min after injection, revealed significantly less intact substrate in the plasma of insulin-treated wildtype mice compared with saline treated wildtype mice. This suggests that insulin increased the clearance of circulating vasopressin, most likely due to a higher proportion of IRAP found at the cell surface following insulin stimulation. Importantly, there was no difference in the clearance of radiolabeled vasopressin from the circulation of saline- or insulin-treated IRAP knockout mice, validating that the vasopressin cleavage in wildtype animals can most likely be attributed to IRAP’s aminopeptidase activity (Wallis et al., 2007). Additionally, these results were not due to insulin-induced changes in total distribution volume or excretion, with similar plasma concentrations of radiolabeled inulin in saline- and insulin-treated wildtype and IRAP knockout mice (Wallis et al., 2007). Taken together, these findings show that increased translocation of IRAP to the cell surface following insulin stimulation, mediated the increase in vasopressin cleavage in wildtype animals. This highlights the physiological role of the N-terminus of IRAP in potentially regulating its aminopeptidase activity at the C-terminus.

## Conclusion

Whilst the catalytic activity of IRAP as well as the mechanisms underlying the trafficking of IRAP-containing vesicles have been independently studied, the influence of one functional domain on the other remains poorly understood. The fact that IRAP alone in the M1 aminopeptidase family possesses trafficking motifs in its N-terminal domain suggests that tight regulation of its localization must be important in determining its functional significance. Additionally, mobilization of IRAP to the cell surface in various pathophysiological states further supports the idea that its localization may reflect the cellular demand for its catalytic activity in a given location and time and may provide a means by which the cell can regulate the degradation of extracellular substrates. These observations provide insights into the interplay between the catalytic activity of IRAP and its trafficking, highlighting the importance of addressing these functions together in future studies.

## Author Contributions

AV wrote the manuscript with input on ideas and writing from SC. TG and PT provided important feedback on the final manuscript. All authors contributed to the article and approved the submitted version.

## Conflict of Interest

The authors declare that the research was conducted in the absence of any commercial or financial relationships that could be construed as a potential conflict of interest.
